# Identification of Cell‐Specific Differential DNA Methylation Associated With Methotrexate Treatment Response in Rheumatoid Arthritis

**DOI:** 10.1002/art.42464

**Published:** 2023-05-09

**Authors:** Cameron Adams, Nisha Nair, Darren Plant, Suzanne M. M. Verstappen, Hong L. Quach, Diana L. Quach, Alex Carvidi, Joanne Nititham, Mary Nakamura, Jonathan Graf, Anne Barton, Lindsey A. Criswell, Lisa F. Barcellos

**Affiliations:** ^1^ School of Public Health, University of California Berkeley; ^2^ Centre of Genetics and Genomics Versus Arthritis, Manchester Academic Health Sciences Centre, The University of Manchester Manchester UK; ^3^ Centre of Genetics and Genomics Versus Arthritis, Manchester Academic Health Sciences Centre, NIHR Manchester BRC, Manchester University Foundation Trust, The University of Manchester Manchester UK; ^4^ NIHR Manchester BRC, Manchester University Foundation Trust, and Centre for Epidemiology Versus Arthritis, Division of Musculoskeletal and Dermatological Sciences, Manchester Academic Health Sciences Centre, The University of Manchester, Manchester, UK, Institute of Cellular Medicine, Newcastle University, and NIHR Newcastle BRC, Newcastle upon Tyne Hospitals NHS Foundation Trust Newcastle upon Tyne UK; ^5^ University of California San Francisco; ^6^ National Human Genome Research Institute, NIH Bethesda Maryland; ^7^ University of California and San Francisco Veterans Administration Health System San Francisco California; ^8^ School of Public Health, University of California, Berkeley, and National Human Genome Research Institute, NIH Bethesda Maryland

## Abstract

**Objective:**

We undertook this study to estimate changes in cell‐specific DNA methylation (DNAm) associated with methotrexate (MTX) response using whole blood samples collected from rheumatoid arthritis (RA) patients before and after initiation of MTX treatment.

**Methods:**

Patients included in this study were from the Rheumatoid Arthritis Medication Study (n = 66) and the University of California San Francisco Rheumatoid Arthritis study (n = 11). All patients met the American College of Rheumatology RA classification criteria. Blood samples were collected at baseline and following treatment. Disease Activity Scores in 28 joints using the C‐reactive protein level were collected at baseline and after 3–6 months of treatment with MTX. Methylation profiles were generated using the Illumina Infinium HumanMethylation450 and MethylationEPIC v1.0 BeadChip arrays using DNA from whole blood. MTX response was defined using the EULAR response criteria (responders showed good/moderate response; nonresponders showed no response). Differentially methylated positions were identified using the Limma software package and Tensor Composition Analysis, which is a method for identifying cell‐specific differential DNAm at the CpG level from tissue‐level (“bulk”) data. Differentially methylated regions were identified using Comb‐p software.

**Results:**

We found evidence of differential global methylation between treatment response groups. Further, we found patterns of cell‐specific differential global methylation associated with MTX response. After correction for multiple testing, 1 differentially methylated position was associated with differential DNAm between responders and nonresponders at baseline in CD4+ T cells, CD8+ T cells, and natural killer cells. Thirty‐nine cell‐specific differentially methylated regions associated with MTX treatment response were identified. There were no significant findings in analyses of whole blood samples.

**Conclusion:**

We identified cell‐specific changes in DNAm that were associated with MTX treatment response in RA patients. Future studies of DNAm and MTX treatment response should include measurements of DNAm from sorted cells.

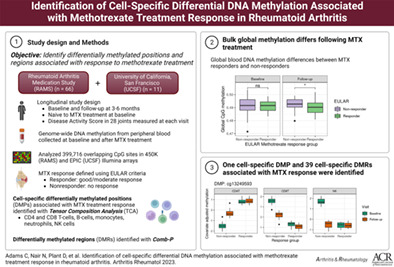

## INTRODUCTION

Rheumatoid arthritis (RA) is the most common systemic autoimmune disease and affects up to 1% of the global population (1). Methotrexate (MTX) is a disease‐modifying antirheumatic drug (DMARD) that is the most frequent first‐line DMARD for the treatment of RA (2). Approximately 30–40% of patients continue with MTX treatment after 2 years (3, 4). Reasons for discontinuation of treatment include inefficacy and adverse events. Significant joint damage can occur in the early phase of RA, and response to the first treatment regimen is an important indicator of long‐term prognosis (5, 6, 7, 8).

MTX is a synthetic folate that has been shown to greatly increase binding affinity for dihydrofolate reductase protein as compared to folic acid (9). The specific mechanisms of the antiinflammatory effects of MTX in the setting of RA are not fully understood but are believed to include the accumulation of adenosine as a result of a reduction in purine metabolism, decreased proliferation and increased apoptosis of immune cells, and inhibition of cytokine production (10). Previous research suggests that medications, including MTX, alter patterns of DNA methylation (DNAm) (9, 11, 12, 13). This is of interest for several reasons, including that treatment‐associated changes in the epigenome may explain, at least in part, the mechanisms of MTX in RA. Further, DNAm patterns prior to treatment and changes in DNAm associated with treatment may serve as predictors of treatment response (14).

The most common tissue type used in epigenome‐wide association studies (EWAS) employing DNAm is blood or peripheral blood mononuclear cells (PBMCs) extracted from blood (15). Peripheral blood contains several different cell types, each of which have different methylation profiles. DNAm measurements taken from blood samples include a combination of DNAm in the constituent cell‐types, and adjustment for global cell proportions is critical when performing EWAS (16, 17). However, adjustment for global cell proportions can limit researchers’ ability to detect differential DNAm, and cell‐specific differential DNAm may be obscured if, for example, the differential DNAm at a CpG for a given phenotype is present in only 1 cell‐type or if the direction of differential DNAm is in opposing directions between certain cell‐types. Using measurements of DNAm in T lymphocytes that were recorded before the initiation of treatment, previous studies identified patterns of global methylation, as well as 2 CpGs that were associated with MTX treatment response (18, 19). Two previous studies investigating the association between DNAm and MTX treatment response using DNAm measurements in whole blood and isolated PBMCs found limited evidence of DNAm associated with MTX response (20, 21). To our knowledge, no studies have investigated DNAm in sorted cells and patient response to treatment with MTX.

In this study, we estimated changes in cell‐specific DNAm associated with treatment response using whole blood samples collected from RA patients before and after initiation of MTX treatment. RA patients included in this study were from the Rheumatoid Arthritis Medication Study (RAMS) and the University of California San Francisco Rheumatoid Arthritis (UCSF‐RA) treatment response study.

## PATIENTS AND METHODS

### Patient data

**Figure 1:**
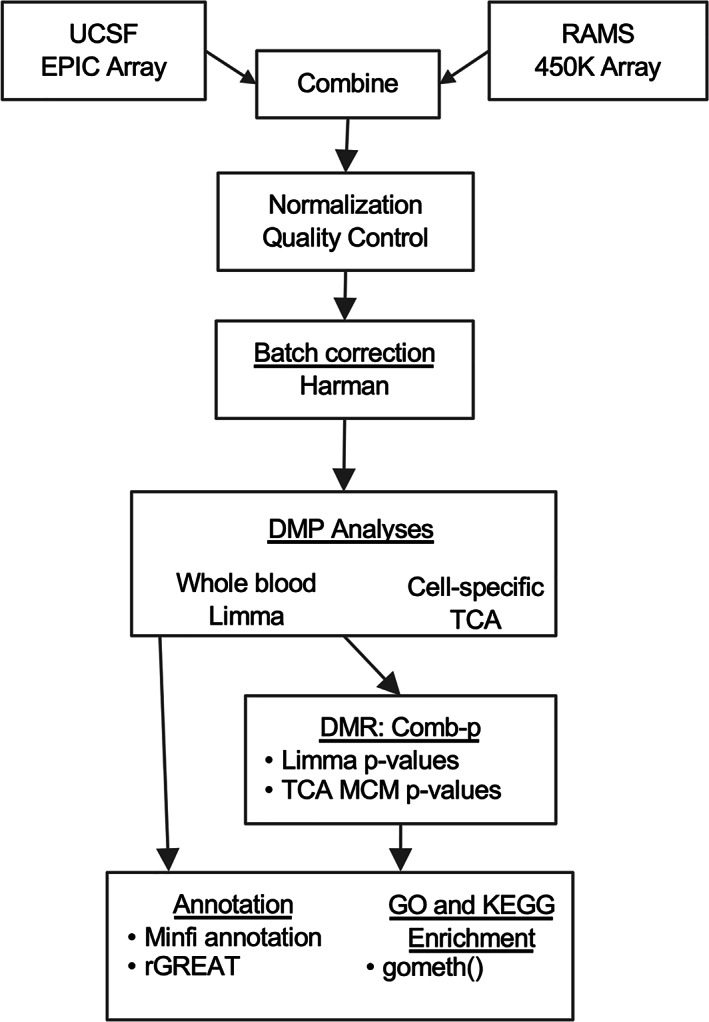
Flow chart illustrating the quality control procedures and differential DNA methylation analyses used in this study of DNA methylation and response to treatment with methotrexate in rheumatoid arthritis patients. UCSF = University of California San Francisco; RAMS = Rheumatoid Arthritis Medication Study; DMP = differentially methylated position; TCA = tensor composition analysis; DMR = differentially methylated region; MCM = marginal conditional model; GO = gene ontology.

A flow chart of study procedures and analyses is displayed in Figure 1. Participants were from the RAMS and the UCSF‐RA study. The methods used in the RAMS have been described previously (20). Briefly, the RAMS is a 1‐year, UK‐based multicenter longitudinal observational study of RA patients and MTX treatment. UCSF‐RA study participants were recruited from rheumatology clinics in San Francisco County between 2016 and 2020. Research protocols for the RAMS and the UCSF‐RA were approved by the research ethics boards at the Central Manchester NHS Research Ethics Committee (project no. 08/H1008/25) and the UCSF Human Research Protection Program (project no. 15‐17175), respectively, and carried out in conformity with the Declaration of Helsinki. Written informed consent was obtained from each patient. Patients from the UCSF‐RA study and the RAMS included in this research were naive to MTX at baseline. For RAMS participants, blood samples were collected at baseline and 4 weeks after treatment initiation. Clinical data and disease activity scores were measured at baseline before treatment initiation and at 6 months after treatment initiation. For UCSF‐RA study participants, blood samples were collected at baseline and at the next follow‐up visit, ~3–6 months after treatment initiation. All patients met the American College of Rheumatology RA classification criteria (22).

### Disease activity scores and treatment response criteria

For this study, the disease activity score based on 28 joints using the C‐reactive protein (DAS28) level was used as the primary measure of disease activity (23). Component measurements of the DAS28 were performed at the pretreatment baseline visit and at the follow‐up visit (6 months after baseline for RAMS participants and 3–6 months after baseline for UCSF‐RA study participants). CRP levels were measured in serum samples collected from whole blood that had been obtained from participants at each visit. Missing DAS28 scores for 2 UCSF‐RA participants were imputed using their Clinical Disease Activity Index scores (24). Response to MTX was defined according to the EULAR response criteria: good response defined as DAS28 ≤3.2 units after treatment and DAS28 improvement >1.2 units; moderate response defined as DAS28 between 3.2 and 5.1 units after treatment and DAS28 improvement between 0.6 and 1.2 units; and no response defined as having DAS28 after treatment >5.1 units and DAS28 improvement ≤0.6 units (25).

### Methylation data quality control

Genome‐wide DNAm profiles for each participant were measured with the Illumina 450K (RAMS participants) and EPIC arrays (UCSF‐RA study participants) using DNA extracted from whole blood samples. Stratified random sampling (RAMS participants) or placement of each participant's pretreatment and posttreatment samples on the same array (UCSF‐RA study participants) was used to balance out the number of pretreatment and posttreatment samples on each DNAm array and to prevent batch effects.

### DNAm data processing

DNAm data were processed using the minfi R package (26). Raw DNAm data from the 450K and EPIC arrays were combined including only overlapping CpG sites (452,567 sites). Normalization and quality control steps were performed on the combined DNAm data. We excluded samples with ≥5% detection (0 samples; *P* < 0.01) and CpGs with ≥5% detection (834 sites; *P* < 0.01). CpG sites with annotated SNPs in the single base extension or CpG were excluded (16,130 sites). Previously identified cross‐reactive probes were also excluded (26,854 sites) (27). Finally, CpG sites on sex chromosomes were excluded, leaving 399,716 CpG sites for analyses. Background subtraction and dye‐bias correction were performed using preprocessNoob, and between‐array normalization was performed using preprocessFunnorm.

### Global cell proportion estimation

Global cell type proportions for B cells, CD8+ T cells, CD4+ T cells, monocytes, natural killer (NK) cells, and neutrophils were estimated separately within each array type (450K and EPIC) using estimateCellCounts2 with IDOL Optimized CpGs (28). Input methylation data were normalized using preprocessNoob.

### Correction for array type

There is substantial overlap in CpG coverage between the EPIC and 450K arrays; however, previous studies have revealed differences in global DNAm between these arrays (29). We used Harman correction, a method to correct DNAm measurements for batch effects constrained by the probability of overcorrection, to remove the effect of array type (EPIC versus 450K) on measurements of global DNAm (30). A matrix of normalized and quality controlled M values was used as input with a confidence limit of 95% corresponding to a 95% probability that only batch variation was being removed. Principal components analysis was used to determine if Harman correction was successful (Supplementary Figure 1, available on the *Arthritis & Rheumatology* website at http://onlinelibrary.wiley.com/doi/10.1002/art.42464).

### Differential position analyses

#### Blood methylation

An EWAS was performed using Limma software (31) to examine the association between differential DNAm in blood samples and response to treatment with MTX. EULAR treatment response was collapsed into the binary variable, TR (TR = 1 for good or moderate response according to EULAR criteria, TR = 0 for nonresponse according to EULAR criteria). The primary parameter of interest in this study was the difference in the change in DNAm between follow‐up (time = 1) and baseline (time = 0) between the treatment response groups. The following time interaction model was used to estimate this parameter:

$$\;{\mathrm{CpG}}_{j}\sim \beta_{0}+\beta_{1}TR_{i}+\beta_{2}\mathrm{Tim}e_{i}+\beta_{3}\left({\mathrm{TR}}_{i}\times \mathrm{Tim}e_{i}\right)$$

where β_3_ is the estimate of the difference in DNAm at CpG_j_ between treatment responders (TR = 1) and nonresponders (TR = 0) between baseline and follow‐up visits. To estimate differences in DNAm between treatment response groups before and after treatment and within treatment response groups over time, we implemented the following models: 1) change in DNAm from baseline to follow‐up among all participants; 2) change in DNAm from baseline to follow‐up separately among treatment responders and nonresponders; 3) change in DNAm at baseline between treatment responders and nonresponders; and 4) change in DNAm at follow‐up between treatment responders and nonresponders. Covariates in all models included age, sex, tobacco smoking history (ever or never smoked), array slide, and estimated global cell proportions. *P* values were adjusted for multiple testing using the Benjamini‐Hochberg method (32). Additionally, due to the limited sample size, CpGs with *P* values less than 1 × 10^−6^ were considered to have suggestive evidence for differential DNAm.

#### Cell‐specific analyses

Tensor Composition Analysis (TCA) was used to estimate differential DNAm between treatment response and cell‐specific DNAm. TCA is a method for estimating cell type–specific associations between DNAm and disease phenotypes using bulk DNAm data (33). The linear models defined above were also implemented in TCA. Estimated global cell proportions were included as input. We implemented the following 2‐step pipeline to estimate differential DNAm in TCA: 1) a joint model that tests for evidence of differential DNAm within any cell type at a CpG; and 2) a marginal conditional model, which tests for evidence of differential DNAm within a particular cell type adjusted for the other cell types at a CpG. The joint model can be thought of as an analysis of variance test and provides evidence for differential DNAm in at least 1 cell type. Joint model *P* values were adjusted for multiple testing using the Benjamini‐Hochberg method (32). All CpGs with *P* values less than 0.05 after correction for multiple testing were tested for cell‐specific differential DNAm using the marginal conditional model. Marginal conditional model *P* values less than 0.05 after correction for multiple testing were considered significant.

### Differential region analyses

Exploratory differentially methylated region (DMR) analyses were performed using the Comb‐p software implemented in the ENmix R package (34, 35). Comb‐p is a moving‐averages method that uses autocorrelation between adjacent *P* values within a genomic window to identify regions of differential DNAm. This method is agnostic to the statistical test used to generate the CpG level *P* values. We identified DMRs using *P* values estimated in Limma and the marginal conditional model test in TCA. To generate genome‐wide *P* values for cell‐specific DMR analyses, marginal conditional models were applied to all CpGs in TCA. The following parameter settings were used: bin.size = 310, seed = 0.001, dist.cutoff = 750bp. The Benjamini‐Hochberg and Dunn–Šidák methods were used to correct for multiple testing (32). DMRs with fewer than 2 CpGs were excluded from the results.

### Whole blood global methylation estimates

Global DNAm in whole‐blood samples was estimated by taking the mean methylation value across all CpG sites that passed quality control in each sample. Differences in global DNAm between response groups at baseline and follow‐up were estimated using *t*‐tests, and statistical significance was assessed using permutation testing (1,000 iterations). Statistical testing of cell‐specific global methylation was not possible using TCA methods; however, bar plots were used to identify evidence for differences in global methylation

### Annotation of DMPs and DMRs

DMPs and DMRs were annotated to genic features and CpG island location using Illumina and UCSC Genome Browser gene annotation. Additionally, the distance to the nearest upstream and downstream transcription start sites for CpGs was annotated using rGREAT (36).

### Pathway analysis and sensitivity analysis

Analyses for enrichment of gene ontology and KEGG pathways were performed using gometh from the missMethyl R package separately for the top 1,000 nominally significant CpGs identified in Limma (*P* < 0.05) and TCA (marginal conditional model *P* < 0.05) analyses. DMP analyses conducted in Limma and TCA were performed separately among participants who self‐identified as White. Pearson's correlation coefficient was used to assess the consistency of estimated changes in DNAm among all participants and among participants identifying as White. To assess the effects of heterogeneity between the UCSF‐RA study data set and the RAMS data set, Limma analyses of DMPs were performed separately within each data set and combined using random effect meta‐analysis. Meta‐analyses could not be performed for TCA analyses because the sample size was too small in the UCSF‐RA study to run TCA. To assess whether CpGs in the top DMPs and DMRs were associated with the data set or methylation array (UCSF‐RA/EPIC array versus RAMS/Illumina 450K array) or self‐reported race, principal components analysis was performed on Harman batch‐corrected methylation measurements for CpGs within the top DMPs and DMRs. Plots were used to assess whether principal components from CpGs in DMPs and DMRs clustered by data set/array or self‐reported race. All statistical analyses were conducted using R version 4.0.2 (37).

## RESULTS

### Study participant characteristics

Baseline characteristics of study participants are reported in Table 1. Thirty‐six RAMS participants and 4 UCSF‐RA study participants were identified as responders to treatment with MTX. Participant ages at baseline were similar between response groups among RAMS participants; however, responders in the UCSF‐RA study were ~ 6 years older than nonresponders. All RAMS participants self‐reported as White. Approximately 50% of the UCSF‐RA study participants were White, with 3 participants identifying as African American, 1 participant identifying as Asian, and 1 participant identifying as Other. Baseline DAS28 scores were similar among the responders in both cohorts. Nonresponders in the RAMS (mean ± SD 4.10 ± 1.3) had a higher baseline DAS28 score than their UCSF‐RA study counterparts (mean ± SD 3.15 ± 1.04).

**Table 1:** Baseline characteristics of participants with rheumatoid arthritis in a study of the effect of methotrexate treatment on DNA methylation*

	RAMS (n = 66)		UCSF‐RA (n = 11)	
	Response (n = 36)†	No response (n = 30)‡	Response (n = 4)†	No response (n = 7)‡
Female	25 (69.4)	24 (80.0)	4 (100.0)	6 (85.7)
Age at baseline, mean ± SD years	60.13 ± 14.02	59.17 ± 15.24	61.85 ± 10.48	54.95 ± 12.05
Self‐reported race				
Asian	0 (0.0)	0 (0.0)	0 (0.0)	1 (14.3)
African American	0 (0.0)	0 (0.0)	1 (25.0)	2 (28.6)
Other	0 (0.0)	0 (0.0)	0 (0.0)	2 (28.6)
White	36 (100.0)	30 (100.0)	3 (75.0)	2 (28.6)
Baseline DAS28‐CRP score, mean ± SD	4.96 ± 1.02	4.10 ± 1.30	3.83 ± 0.70	3.15 ± 1.04
Ever smoker, yes	20 (55.6)	16 (53.3)	1 (25.0)	5 (71.4)

* Except where indicated otherwise, values are the number (%) or participants. RAMS = Rheumatoid Arthritis Medication Study; UCSF‐RA = University of California, San Francisco Rheumatoid Arthritis Study; DAS28 = Disease Activity Score in 28 joints using the C‐reactive protein level.

† Treatment response was defined as “good” or “moderate” according to the EULAR response criteria.

‡ Treatment response was defined as “none” according to the EULAR response criteria.

### Differences in global methylation between treatment responders and nonresponders

**Figure 2:**
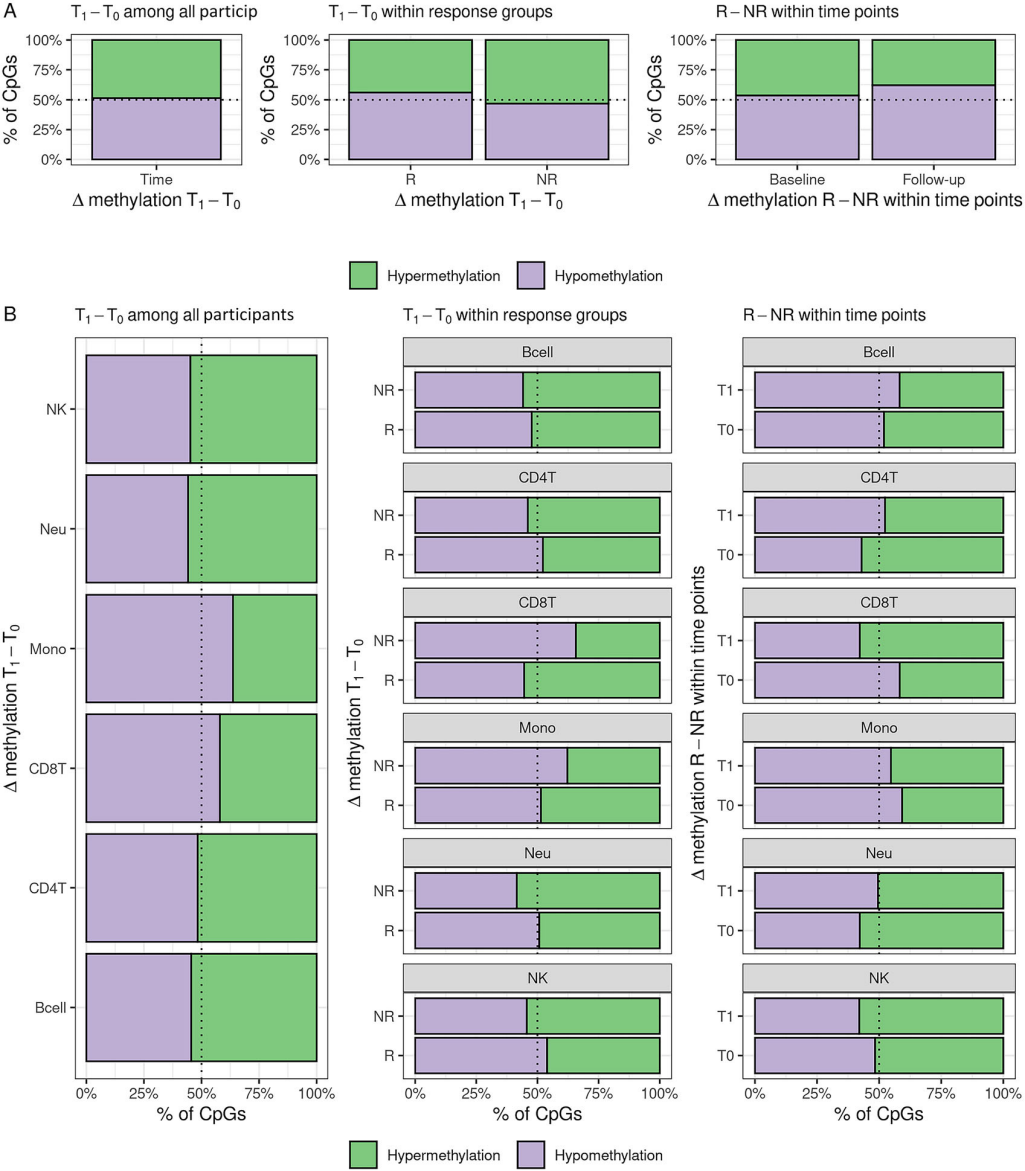
Distribution of direction of estimated differential DNA methylation. (**A**) Whole blood Limma models and (**B**) cell‐specific TCA models. R = treatment response; NR = treatment nonresponse; T_0_ = DNA methylation at baseline visit; T_1_ = DNA methylation at follow‐up visit; NK = natural killer; Neu = neutrophil; Mono = monocyte; CD8T = CD8+ T cell; CD4T = CD4+ T cell; Bcell = B cell.

The distribution of differential DNAm estimates from Limma and TCA analyses are presented in Figure 2. Among all participants, ~50% of CpGs were hypomethylated (Figure 2A) following treatment. The direction of global DNAm was different between MTX treatment responders and nonresponders (based on the EULAR response criteria). Average DNAm following treatment among treatment responders was reduced compared to nonresponders. We found reduced methylation among responders (49.01%) compared to nonresponders (49.25%) at the follow‐up visit (permutation test *P* = 0.022) but not at baseline (permutation test *P* = 0.77) (Supplementary Figure 2, http://onlinelibrary.wiley.com/doi/10.1002/art.42464).

### Cell‐specific changes in global methylation

Based on the visual inspection of bar plots, there was evidence that the proportion of CpGs that were hypomethylated following treatment with MTX were different between cell‐types (Figure 2B). The direction of DNAm change following MTX treatment was different between treatment response groups. For example, CPG sites in neutrophils were more likely to be hypomethylated in nonresponders than in responders. This contrasted with DNAm changes in monocytes, where there was more hypermethylation in nonresponders than in responders. These cell‐specific differences were also seen between treatment response groups within each visit.

### DMP results

Five CpGs demonstrated *P* values less than 1 × 10^−6^ in Limma analyses of DMPs (Table 2). Two CpGs (cg06336912 and cg15936718) were associated with decreased DNAm at the follow‐up visit in MTX treatment responders compared to nonresponders. The other 3 CpGs were from the model comparing change in DNAm over time between responders and nonresponders (cg16868591), change in DNAm over time among responders (cg19506849), and change in DNAm among nonresponders (cg18224793).

**Table 2:** Results of Limma analysis of differentially methylated positions*

		Annotation				
CpG	chr:bp (hg19)	GREAT†	UCSC gene	Model	% Δβ	*P*
cg06336912	chr16:90173019	*PRDM7* (–30,681)		T_1_: R–NR	–5.86	1 × 6.5^−6^
cg15936718	chr16:90088801	*GAS8* (–207)	*GAS8*	T_1_: R–NR	–3.49	1 × 8.9^−6^
cg16868591	chr19:12803493	*DHPS* (–10,777); *FBXW9* (+3,964)	*FBXW9*	(R_1_–R_0_)– (NR_1_–NR_0_)	–3.39	1 × 3.0^−6^
cg19506849	chr10:114767609	*HABP2* (–545,176); *TCF7L2* (+57,600)	*TCF7L2*	R_1_–R_0_	2.64	1 × 9.4^−6^
cg18224793	chr7:100222124	*MOSPD3* (+11,991); *TFR2* (+18,220)	*TFR2*	NR_1_–NR_0_	2.34	1 × 4.7^−6^

* Five CpGs demonstrated significance (*P* < 1 × 10^−6^) in Limma analyses of differentially methylated positions. chr = chromosome; GREAT = Genomic Regions Enrichment of Annotations Tool; NR = nonresponder; R = responder; R_0_ = responder at baseline visit; R_1_ = responder at follow‐up visit; NR_0_ = nonresponder at baseline visit; NR_1_ = nonresponder at follow‐up visit; T_1_ = follow‐up visit.

† Distance from CpG to transcription start site of nearest upstream and downstream gene annotated using rGREAT software.

One DMP reached significance after correction for multiple testing in the TCA joint models (Table 3). Cg13249593 was associated with cell‐specific differential DNAm between responders and nonresponders at baseline in CD4+ T cells, CD8+ T cells, and NK cells. The site cg13249593 is located within a CpG island ~21 kb upstream of *KRT19* and 23 kb downstream of *KRT9* and is a predicted enhancer (GH17J041546) for several genes, including *KRT19*, *KRT9*, *SMARCE1*, and *CAVIN1* (Supplementary Figure 3 and Supplementary Table 1, http://onlinelibrary.wiley.com/doi/10.1002/art.42464) (38).

**Table 3:** Results of the tensor composition analysis of differentially methylated positions

CpG	chr:bp (hg19)	GREAT annotation*	Model†	Cell type	Δ Methylation	Joint model *P* ‡	Marginal conditional model *P*
cg13249593	chr17:39705234	KRT19 (–20,674); KRT9 (+23,076)	T_0_: R–NR	CD4T CD8T	0.226–0.310	1 × 2.4^−8^ 1 × 9.5^−3^ §	1 × 4.6^−6^ 1 × 1.8^−5^
				NK	–0.489		1 × 2.3^−6^

* Distance from CpG to transcription start site of nearest upstream and downstream gene annotated using rGREAT software. See Table 2 for definitions.

† Model comparing difference in DNA methylation at baseline before treatment with methotrexate between rheumatoid arthritis patients identified as treatment responders compared to nonresponders.

‡ Joint model in tensor composition analysis tested for evidence of differential DNA methylation within any cell type at each CpG.

§ *P* for false discovery rate.

### DMR results

No DMRs were identified using Limma analyses. Using *P* values from TCA marginal conditional models, 39 cell‐specific DMRs were identified in models comparing change in DNAm over time among nonresponders (3 DMRs), change in DNAm over time among responders (3 DMRs), and change in DNAm between responders and nonresponders at baseline (16 DMRs) and at follow‐up (17 DMRs). The top 10 DMRs ranked by *P* value are presented in Table 4. The top DMRs were located close to transcription start sites and CpG islands (see Supplementary Table 2 for complete DMR results, http://onlinelibrary.wiley.com/doi/10.1002/art.42464).

**Table 4:** Top 10 cell‐specific differentially methylated regions determined using tensor composition analysis*

Region, chr:bp	Model	Cell type	No. CpG	Δ Methylation	*P*	GREAT annotation	Illumina annotation		
						Gene†	Gene	Feature	CpG island
1:161008461–61008826	T_0_: R–NR	CD8T	8	Hyper	1 × 9.5^−18^	*TSTD1* (+136)	*TSTD1*	TS200	Island
12:9217389–9217907	T_0_: R–NR	Bcell	10	Hyper	1 × 5.8^−15^	–	*LOC144571*	TS200	Island
1:202310823–202311278	T_1_: R–NR	Neu	7	Hyper	1 × 3.1^−14^	*UBE2T* (+57)	*UBE2T*	TS200	Island
17:6899084–6899577	T_1_: R–NR	Mono	11	Hyper	1 × 2.0^−13^	*ALOX12* (–53)	*ALOX12*	TS200	Island
13:36871753–36872346	R: T_1_–T_0_	NK	9	Hypo	1 × 8.6^−13^	–	*C13orf38*	TS200	Island
1:161008461–161008826	T_0_: R–NR	CD4T	8	Hypo	1 × 1.8^−12^	*TSTD1* (+136)	*TSTD1*	TS200	Island
17:6899084–6899577	T_1_: R–NR	NK	11	Hypo	1 × 4.9^−12^	*ALOX12* (–53)	*ALOX12*	TS200	Island
10:77542301–77542585	T_0_: R–NR	CD4T	9	Hyper	1 × 1.5^−11^	*C10orf11* (–76)	*C10orf11*	TS200	OpenSea
6:32063725–32064258	T_1_: R–NR	NK	16	Hypo	1 × 5.9^−11^	*TNXB* (–50,087)	*TNXB*	Body	Island
6:31148331–31148748	T_0_: R–NR	CD8T	15	Hyper	1 × 1.2^−10^	–	–	–	Island

* GREAT = Genomic Regions Enrichment of Annotations Tool; chr = chromosome; Hyper = hypermethylation; Hypo = hypomethylation; NR = nonresponder; R = responder; T_0_ = baseline visit.

† Distance from CpG to transcription start site of the nearest upstream and downstream gene annotated using rGREAT software.

### Pathway analyses

No gene ontology pathways reached significance after correction for multiple testing; however, several gene ontologies that reached nominal significance in cell‐specific differential CpGs from TCA analyses are related to immune function and MTX response (Supplementary Figure 4, http://onlinelibrary.wiley.com/doi/10.1002/art.42464). At baseline, CpGs associated with differential DNAm between MTX treatment nonresponders were enriched for pathways related to regulation of the immune system, adaptive immune response, and lymphocyte differentiation, among others. After treatment, CpGs associated with differential DNAm between treatment response groups were enriched for pathways possibly related to MTX response (antiporter activity and regulation of purine nucleotide metabolic process) and lymphocyte differentiation and proliferation.

In the KEGG pathway analyses, the Wnt signaling pathway (hsa04310; *P* = 8.9 × 10^−5^, false discovery rate–corrected *P* = 0.03) was significant after correction for multiple testing in Limma analyses estimating differential DNAm over time among all participants.

### Comparison of DNAm estimates with previous studies

In a study by Glossop et al, 2 CpGs in T lymphocytes (cg03018489 and cg14345882) were found to be associated with MTX treatment response as measured by the DAS28 at baseline before treatment initiation (18). In Limma analyses, cg14345882 was associated with hypermethylation in responders compared to nonresponders at baseline at nominal significance (Δβ = 6.1%; *P* = 3.1 × 10^−3^). In results from TCA, the estimated direction of differential DNAm at cg14345882 was the same in CD4+ and CD8+ T cells as reported by Glossop et al., but was not significant (*P* > 0.05). The CpG cg03018489 was excluded during quality control. In a study by Gosselt et al, evidence was found of differences in global DNAm in leukocytes between treatment response groups, with higher DNAm associated with treatment nonresponse (39). In our DNAm data from whole blood samples, we found evidence of differences in global DNAm between MTX responders and nonresponders after treatment (β = –0.24%; *P* = 0.03) but not at baseline (β = –0.03%; *P* = 0.79). Estimated cell‐specific differential DNAm between treatment responders and nonresponders from TCA models indicates increased DNAm among nonresponders at baseline in CD8+ T cells and monocytes, and increased DNAm among responders at baseline in CD4+ T cells and neutrophils (Figure 2B).

### Sensitivity analyses

Estimated changes in DNAm from analyses restricted to participants who self‐identified as White were strongly correlated with those estimated in all participants in both the Limma and TCA analyses (Supplementary Figures 5 and 6, http://onlinelibrary.wiley.com/doi/10.1002/art.42464). Estimated coefficients were also similar in magnitude and direction in the most significant DMPs, and *P* values were generally similar in magnitude (Supplementary Tables 3 and 4, http://onlinelibrary.wiley.com/doi/10.1002/art.42464). Results from meta‐analyses were consistent with estimated coefficients in the top DMPs in Limma analyses (Supplementary Table 5 and Supplementary Figure 7, http://onlinelibrary.wiley.com/doi/10.1002/art.42464). Estimates of heterogeneity (I^2^ and Cochran's Q statistic) were not significant. Plots of the first 6 components from principal components analysis of CpGs in DMPs and DMRs (reported in Tables 2, 3, and 4 and Supplementary Table 2, http://onlinelibrary.wiley.com/doi/10.1002/art.42464) did not show evidence of clustering by data set/array type or by self‐reported race (Supplementary Figure 8, http://onlinelibrary.wiley.com/doi/10.1002/art.42464).

## DISCUSSION

In this study, we investigated differential DNAm associated with EULAR treatment response to MTX among MTX‐native RA patients. This is the first study to report results for cell‐specific differential DNAm estimated using whole blood samples in studies of MTX response in RA patients. Similar to recent studies investigating differential DNAm and response to MTX treatment, no significant DMPs or DMRs associated with treatment response were identified after correcting for multiple testing in analyses of whole blood (20, 21). One DMP and 39 DMRs from cell‐specific analyses were identified after correction for multiple tests. The site cg13249593 was associated with differential DNAm in treatment responders compared to nonresponders at baseline before treatment in CD4+ (hyper) T cells, CD8+ (hypo) T cells, and NK (hypo) cells. It is located approximately 21 kb upstream and 23 kb downstream of the transcription start sites of *KRT9* and *KRT19*, respectively, on chromosome 17. *KRT19* produces the protein keratin 19, which has been found in the synovial fluid of RA patients. Keratin 19 has been found to be an autoantigen in anti–cyclic citrullinated peptide–positive RA patients (40). Evidence for differential DNAm at cg13249593 was found in the Limma analyses as well (Δβ = –0.37; *P* = 1.5 × 10^−3^) but was not significant after correction for multiple tests. Estimated coefficients from Limma and TCA indicate that treatment with MTX was associated with DNAm changes in nonresponders to levels observed in responders and had no discernible effect in responders (Supplementary Figure 9, http://onlinelibrary.wiley.com/doi/10.1002/art.42464).

The site cg13249593 is in a CpG island and is in a region that is a predicted enhancer for several nearby genes; data from the NIH Roadmap Epigenomics Mapping Consortium shows that cg13249593 is located in an enhancer or flanking promoter region, depending on the cell type (CD4+, CD8+, or NK cells; Supplementary Figure 3, http://onlinelibrary.wiley.com/doi/10.1002/art.42464). The differences in DNAm at baseline between the treatment response groups suggest that expression of genes influenced by DNAm levels at cg13249593 may be different between treatment response groups; however, response to treatment with MTX was not associated with the changes in DNAm at cg13249593 observed in nonresponders. The top DMR analyses identified several cell‐specific DMRs. Of note, a DMR located 53 bp from the transcription start site of *ALOX12* was associated with differential DNAm between responders and nonresponders at follow‐up in monocytes and NK cells. Previous research has found that increased expression of *ALOX12* in monocytes is associated with juvenile RA and that MTX decreases the expression of *ALOX12* (41, 42). Estimated coefficients from TCA indicate that DNAm was higher among MTX treatment responders than in nonresponders, suggesting decreased expression among responders.

There was evidence of differential DNAm in the top 20 genes associated with MTX response from the Comparative Toxicogenomics Database (41) in Limma and TCA analyses at *P* < 0.05 (Supplementary Figure 10, http://onlinelibrary.wiley.com/doi/10.1002/art.42464). These genes included *SLC19A1*, the gene most strongly associated with MTX response, as well as *DHFR* and *BLC2*. *SLC19A1* is a folate transporter and is integral to the MTX pathway (43). Inhibition of *DHFR* expression through the increased binding affinity of MTX (a synthetic folate) for *DHFR*, compared to folate, is the primary mechanism of MTX in the treatment of cancer (9). *BCL2* is an apoptosis regulator, and inhibition of B cell apoptosis has been associated with increased expression of *BCL2* in RA patients (44).

Our results are consistent with findings from a previous study by Glossop et al, which identified 2 CpGs predictive of MTX response before treatment in T lymphocytes (18). While our results did not reach genome‐wide significance, the direction of differential DNAm at the CpG that passed quality control, both in blood and in CD4+ and CD8+ T cells, were the same as those reported by Glossop et al, with higher baseline DNAm among MTX responders than nonresponders (Supplementary Figure 10, http://onlinelibrary.wiley.com/doi/10.1002/art.42464). We were not able to replicate the findings of Gosselt et al with regard to the differences in global DNAm between response groups at baseline; however, we did find evidence of differential global DNAm in whole blood samples after treatment. We did not have cell‐specific measurements of DNAm and therefore could not estimate global cell‐specific differences in DNAm directly. However, the distribution of hypermethylated versus hypomethylated CpG sites between response groups at baseline and follow‐up in the TCA analyses provides evidence of differences in DNAm at baseline between the response groups, specifically in CD4+ T cells, CD8+ T cells, monocytes, and neutrophils.

The lack of significant DMPs and DMRs in whole blood samples in this study is similar to the results of 2 previous studies which investigated response to DNAm and did not find evidence that differential DNAm was associated with MTX treatment response. Recent studies demonstrating evidence of an association between differential DNAm and MTX response used DNAm quantified in T lymphocytes (18). This finding, along with our findings of differences in the patterns of global cell‐specific DNAm between MTX treatment response groups and the identification of a cell‐specific DMP and several cell‐specific DMRs, suggests that associations with DNAm and MTX response are cell‐type specific.

Strengths of this study included the prospective design used for each data set. Patients in the RAMS and the UCSF‐RA study were naive to MTX at baseline, and blood samples and disease activity scores were collected both before and after the initiation of treatment. Furthermore, all participants received MTX monotherapy rather than combination therapy during the observation period. To our knowledge, this is the first study to report on cell‐specific DNAm associated with MTX using methods to estimate cell‐specific DNAm from whole blood samples. Patients in the RAMS reported treatment compliance >80% (20). We were unable to evaluate MTX treatment compliance in the UCSF‐RA study participants.

Limitations of this study included residual confounding resulting from combining DNAm data from the Illumina 450K and EPIC platforms; however, results from principal components analyses indicate that we were able to remove array effects with the Harman correction (Supplementary Figures 1 and 7, http://onlinelibrary.wiley.com/doi/10.1002/art.42464). Other limitations of this study included a lack of diversity with regard to participant demographic characteristics and the small sample size. Participants were recruited from rheumatology clinics in the UK and the San Francisco Bay Area, and nearly all participants self‐identified as White, which limits the generalizability of our findings. Furthermore, the sample size was relatively small, which limited the power to identify smaller changes in DNAm with genome‐wide significance.

Other limitations included differences in the study protocols between the RAMS and the UCSF‐RA study. In the RAMS, blood samples used for the analysis of DNAm were collected at baseline and after 4 weeks of treatment, while blood samples from the UCSF‐RA study participants were collected at baseline and the follow‐up visit. There were also some limitations in the TCA analyses. First, the estimates of differential DNAm in TCA were not estimated using measurements of cell‐specific DNAm in our participants. Another limitation was that there is more power to detect differential DNAm in more abundant cell types compared to less abundant cell types. Finally, the 2 time‐point models used in the Limma analysis and TCA were slightly different. The Limma models included a random effect for each participant. The TCA software cannot perform paired analyses using random effects or by including participant IDs as a covariate. Including IDs as a covariate would create an unidentifiable model (no. of cell‐types × no. of parameters in the model).

In conclusion, we estimated changes in DNAm associated with response to treatment with MTX in RA patients through the use of methods that deconvolute cell‐specific DNAm at the CpG level. We identified evidence of cell‐specific differential DNAm between treatment responders and nonresponders at baseline in 1 DMP with genome‐wide significance. We also identified 39 cell‐specific DMRs. No DMPs or DMRs were identified in analyses of whole blood samples after correction for multiple testing, although we were not powered to detect modest effects. Our findings of cell‐specific differential DNAm associated with MTX response and the paucity of evidence of differential DNAm in this present research and in similar studies using DNAm from whole blood samples and PBMC samples indicate that future studies of DNAm and MTX response will require larger sample sizes to detect modest effects of MTX on the methylome and DNAm data derived from sorted cells.

## AUTHOR CONTRIBUTIONS

All authors were involved in drafting the article or revising it critically for important intellectual content, and all authors approved the final version to be published. Dr. Barcellos had full access to all of the data in the study and takes responsibility for the integrity of the data and the accuracy of the data analysis.

### Study conception and design

Adams, Nair, Plant, Verstappen, Nakamura, Graf, Barton, Criswell, Barcellos.

### Acquisition of data

Nair, H. Quach, D. Quach, Carvidi, Criswell, Barcellos.

### Analysis and interpretation of data

Adams, Nair, Criswell, Barcellos.

## Supporting information

Disclosure Form

**Figure S1:** PCA plots of methylation data before and after correction for 450K vs. EPIC platform using Harman.
**Figure S2:** Permuted p‐values for associations between changes in whole blood global methylation and EULAR response.
**Figure S3:** Evidence from (A) the UCSC Genome Browser and (B) Epigenetic Mapping Consortium indicates that cg06336912 is in an predicted enhancer region for several genes and is predicted to be either an enhancer (yellow) or flanking promotor (red) in CD4, CD8, and NK cells.
**Figure S4:** GO pathway analysis results for ontologies related to immune function and MTX response from top 1000 DMPs (*P* < 0.05) for each cell‐type. Abbreviations: R0‐NR0, model for difference in DNA methylation between EULAR responders and non‐responders at baseline; R1‐NR1, model for difference in DNA methylation between EULAR responders and non‐responders at follow‐up; (R1‐ R0)‐ (NR1‐ NR0), Difference in change in DNA methylation over time between treatment responders and non‐responders.
**Figure S5:** Comparison of beta coefficients from limma analyses with all participants (xaxis) and subset to participants self‐reporting as White (y‐axis).
**Figure S6:** Comparison of beta coefficients from TCA analyses with all participants (xaxis) and subset to participants self‐reporting as white (y‐axis).
**Figure S7:** Forest plots of meta‐analysis results for *limma* DMP results with 𝒑 < 𝟏 × 𝟏𝟎!.
**Figure S8:** Principal components analysis of CpGs in DMPs and DMRs stratified by dataset and self‐report race identification.
**Figure S9:** cg13249593 DNA methylation within treatment response groups at baseline and at follow‐up measured in whole blood and in CD4+ and CD8+ T cells and natural killer cells. Methylation values are residuals from models adjusted for sex, age, smoking history, batch, and cell‐type proportions (blood only). Cell‐specific DNA methylation estimated in TCA.
**Figure S10:** Summary of evidence of differential methylation among top 20 genes with evidence of pharmacogenomic interaction with MTX from the Comparative Toxicogenomic Database (http://ctdbase.org/). If more than one CpG was annotated to a gene, the CpG with the minimum p‐value was selected. A) *limma* models, and B) *TCA* models.
**Figure S11:** cg14345882 methylation within treatment response groups at baseline in DNA methylation from whole blood and in CD4+ and CD8+ T‐cells estimated by TCA. Methylation values are residuals from models adjusted for sex, age, smoking history, batch, and cell‐type proportions (blood only).

**Table S‐1** cg13249593 is in predicted enhancer (GH17J041546) for several genes.
**Table S‐2**. *TCA* cell‐specific DMRs.
**Table S‐3**. Results from analyses among self‐report white participants for *limma* DMP results with p < 1 × 10^−6^.
**Table S‐4**. Results from analyses among self‐report white participants for *TCA* DMP analyses.
**Table S‐5**. Meta analysis results for *limma* DMP results with p < 1 × 10^−6^.
