# Supplementary material for: Identification of Cell‐Specific Differential DNA Methylation Associated With Methotrexate Treatment Response in Rheumatoid Arthritis
Source: Arthritis Rheumatol. 2023 May 9;75(7):1088–97. doi: 10.1002/art.42464 (PMC10313739; doi:10.1002/art.42464)
Supplement: Supplementary file 2 — Figure S1: PCA plots of methylation data before and after correction for 450K vs. EPIC platform using Harman. Figure S2: Permuted p‐values for associations between changes in whole blood global methylation and EULAR response. Figure S3: Evidence from (A) the UCSC Genome Browser and (B) Epigenetic Mapping Consortium indicates that cg06336912 is in an predicted enhancer region for several genes and is predicted to be either an enhancer (yellow) or flanking promotor (red) in CD4, CD8, and NK cells. Figure S4: GO pathway analysis results for ontologies related to immune function and MTX response from top 1000 DMPs (P < 0.05) for each cell‐type. Abbreviations: R0‐NR0, model for difference in DNA methylation between EULAR responders and non‐responders at baseline; R1‐NR1, model for difference in DNA methylation between EULAR responders and non‐responders at follow‐up; (R1‐ R0)‐ (NR1‐ NR0), Difference in change in DNA methylation over time between treatment responders and non‐responders. Figure S5: Comparison of beta coefficients from limma analyses with all participants (xaxis) and subset to participants self‐reporting as White (y‐axis). Figure S6: Comparison of beta coefficients from TCA analyses with all participants (xaxis) and subset to participants self‐reporting as white (y‐axis). Figure S7: Forest plots of meta‐analysis results for limma DMP results with 𝒑 < 𝟏 × 𝟏𝟎!. Figure S8: Principal components analysis of CpGs in DMPs and DMRs stratified by dataset and self‐report race identification. Figure S9: cg13249593 DNA methylation within treatment response groups at baseline and at follow‐up measured in whole blood and in CD4+ and CD8+ T cells and natural killer cells. Methylation values are residuals from models adjusted for sex, age, smoking history, batch, and cell‐type proportions (blood only). Cell‐specific DNA methylation estimated in TCA. Figure S10: Summary of evidence of differential methylation among top 20 genes with evidence of pharmacogenomic interaction w [file ART-75-1088-s002.docx]

**Supplementary Figures 1–11.**

**Figure 1**. **PCA plots of methylation data before and after correction for 450K vs. EPIC**

**platform using Harman.**

**Figure 2. Permuted p-values for associations between changes in whole blood global**

**methylation and EULAR response.**

**Figure 3. Evidence from (A) the UCSC Genome Browser and (B) Epigenetic Mapping**

**Consortium indicates that cg06336912 is in an predicted enhancer region for several genes**

**and is predicted to be either an enhancer (yellow) or flanking promotor (red) in CD4, CD8,**

**and NK cells.**

**Figure 4. GO pathway analysis results for ontologies related to immune function and**

**MTX response from top 1000 DMPs (*P*<0.05) for each cell-type.** Abbreviations: R0-NR0,

model for difference in DNA methylation between EULAR responders and non-responders at

baseline; R1-NR1, model for difference in DNA methylation between EULAR responders and

non-responders at follow-up; (R1- R0)- (NR1- NR0), Difference in change in DNA methylation

over time between treatment responders and non-responders.

**Figure 5. Comparison of beta coefficients from limma analyses with all participants (xaxis)**

**and subset to participants self-reporting as White (y-axis).**

**Figure 6. Comparison of beta coefficients from TCA analyses with all participants (xaxis)**

**and subset to participants self-reporting as white (y-axis).**


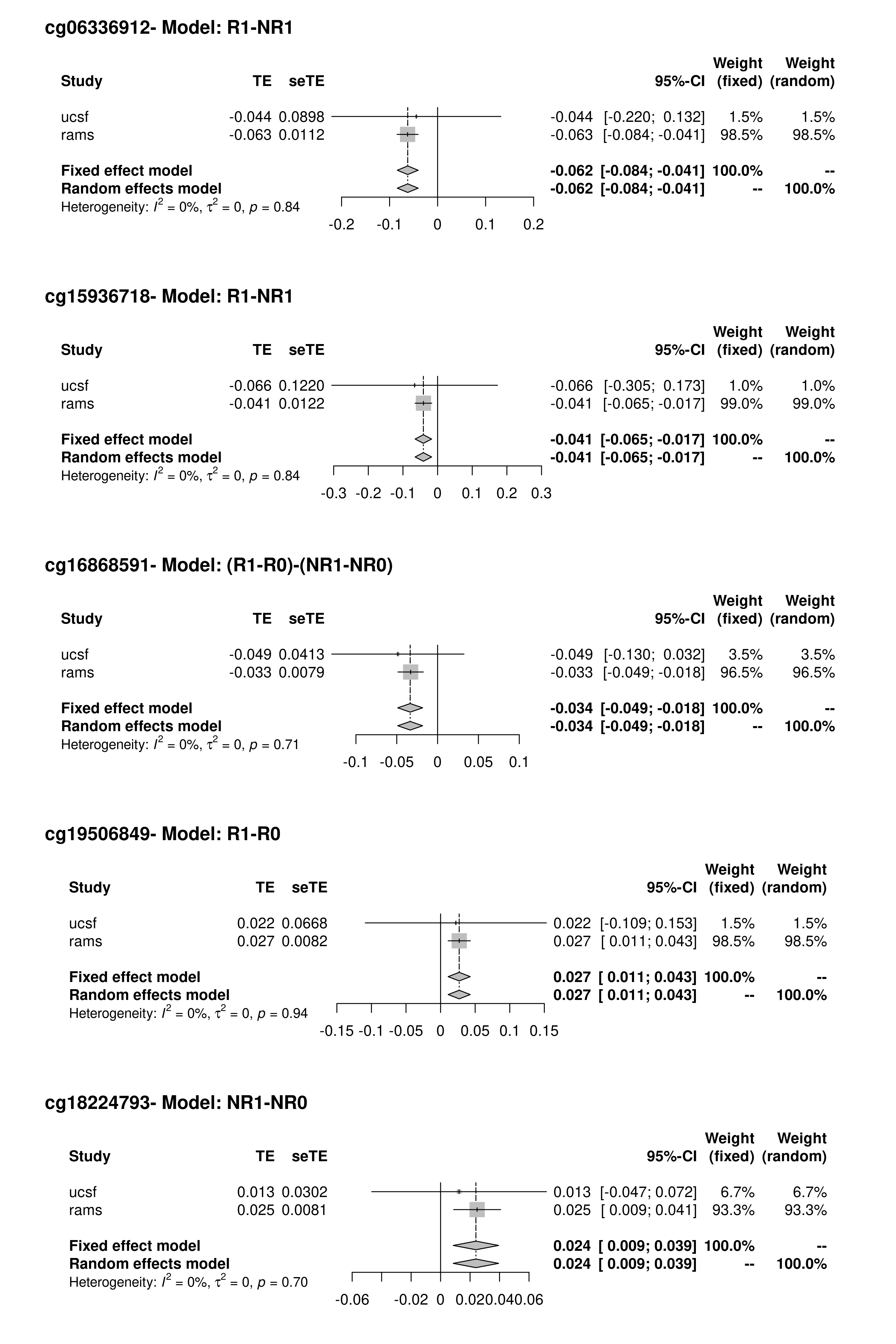


**Figure 7. Forest plots of meta-analysis results for *limma* DMP results with** 𝒑 <

𝟏 × 𝟏𝟎!𝟔**.**

**Figure 8. Principal components analysis of CpGs in DMPs and DMRs stratified by**

**dataset and self-report race identification.**

**Figure 9. cg13249593 DNA methylation within treatment response groups at baseline and**

**at follow-up measured in whole blood and in CD4+ and CD8+ T cells and natural killer**

**cells.** Methylation values are residuals from models adjusted for sex, age, smoking history, batch,

and cell-type proportions (blood only). Cell-specific DNA methylation estimated in TCA.

**Figure 10. Summary of evidence of differential methylation among top 20 genes with**

**evidence of pharmacogenomic interaction with MTX from the Comparative**

**Toxicogenomic Database (http://ctdbase.org/).** If more than one CpG was annotated to a gene,

the CpG with the minimum p-value was selected. A) *limma* models, and B) *TCA* models.

**Figure 11. cg14345882 methylation within treatment response groups at baseline in DNA**

**methylation from whole blood and in CD4+ and CD8+ T-cells estimated by TCA.**

Methylation values are residuals from models adjusted for sex, age, smoking history, batch, and

cell-type proportions (blood only).
