# Supplementary material for: Identification of Cell‐Specific Differential DNA Methylation Associated With Methotrexate Treatment Response in Rheumatoid Arthritis
Source: Arthritis Rheumatol. 2023 May 9;75(7):1088–97. doi: 10.1002/art.42464 (PMC10313739; doi:10.1002/art.42464)
Supplement: Supplementary file 3 — Table S‐1 cg13249593 is in predicted enhancer (GH17J041546) for several genes. Table S‐2. TCA cell‐specific DMRs. Table S‐3. Results from analyses among self‐report white participants for limma DMP results with p < 1 × 10^−6^. Table S‐4. Results from analyses among self‐report white participants for TCA DMP analyses. Table S‐5. Meta analysis results for limma DMP results with p < 1 × 10^−6^. [file ART-75-1088-s003.pdf]

Table S-1. cg13249593 is in predicted enhancer (GH17J041546) for several genes.

Gene	Description	Category	GIFtS	Relevance Score
ACLY	ATP Citrate Lyase	Protein Coding	50	1.07
STAT5A	Signal Transducer And Activator Of Transcription 5A	Protein Coding	50	1.07
KRT14	Keratin 14	Protein Coding	49	1.07
SMARCE1	SWI/SNF Related, Matrix Associated, Actin Dependent Regulator of Chromatin, Subfamily E, Member 1	Protein Coding	47	1.07
KRT19	Keratin 19	Protein Coding	46	1.07
NAGLU	N-Acetyl-Alpha-Glucosaminidase	Protein Coding	45	1.07
KRT9	Keratin 9	Protein Coding	44	1.07
CAVIN1	Caveolae Associated Protein 1	Protein Coding	43	1.07
KRT15	Keratin 15	Protein Coding	41	1.07
KLHL10	Kelch Like Family Member 10	Protein Coding	40	1.07
LINC00974	Long Intergenic Non-Protein Coding RNA 974	RNA Gene	16	1.07
ENSG00000267758	Novel Transcript	RNA Gene	9	1.07
HSALNG0116441		RNA Gene	5	1.07
piR-48749-021		RNA Gene	4	1.07
LOC116276463	CRISPRi-Validated Cis-Regulatory Element Chr17.2497	Biological Region	2	0.93

Abbreviations: GIFtS, GeneCards Inferred Functionality Scores.
Table data from https://www.genecards.org/Search/Keyword?queryString=GH17J041546

Table S-2. *TCA* cell-specific DMRs.

Region chr:bp	Model	Cell type	No. CpG	ΔMeth.	*p*	GREAT annotation[a] Gene (Dist. to TSS)	Illumina annotation Gene	Illumina annotation Feature	Illumina annotation CpG Island
1:161008461_161008826	R0-NR0	CD8T	8	hyper	9.5e-18		TSTD1	TS200	Island
12:9217389_9217907	R0-NR0	Bcell	10	hyper	5.8e-15		LOC144571	TS200	Island
1:202310823_202311278	R1-NR1	Neu	7	hyper	3.1e-14		UBE2T	TS200	Island
17:6899084_6899577	R1-NR1	Mono	11	hyper	2.0e-13	ALOX12 (-53)	ALOX12	TS200	Island
13:36871753_36872346	R1-R0	NK	9	hypo	8.6e-13		C13orf38	TS200	Island
1:161008461_161008826	R0-NR0	CD4T	8	hypo	1.8e-12		TSTD1	TS200	Island
17:6899084_6899577	R1-NR1	NK	11	hypo	4.9e-12	ALOX12 (-53)	ALOX12	TS200	Island
10:77542301_77542585	R0-NR0	CD4T	9	hyper	1.5e-11		C10orf11	TS200	OpenSea
6:32063725_32064258	R1-NR1	NK	16	hypo	5.9e-11	TNXB (-50087)	TNXB	Body	Island
6:31148331_31148748	R0-NR0	CD8T	15	hyper	1.2e-10				Island
13:111267666_111268031	R1-NR1	Bcell	10	hypo	1.1e-10		CARKD	TS200	Island
6:30881463_30881920	R0-NR0	NK	22	hypo	2.0e-10		VARS2	TS200	Island
8:22132664_22133004	R0-NR0	CD8T	9	hypo	2.0e-10		PIWIL2	TS200	Island
12:9217509_9217907	R0-NR0	CD8T	9	hypo	9.9e-10		LOC144571	TS200	Island
19:55549589_55549842	R1-NR1	Mono	7	hypo	9.8e-10		GP6	TS200	OpenSea
6:30419490_30419576	R0-NR0	CD4T	5	hypo	4.0e-10				Island
16:1030387_1030619	R0-NR0	CD4T	7	hyper	4.5e-09	SOX8 (-1305)	SOX8	TSS1500	Island
10:42862875_42863444	R1-NR1	NK	5	hypo	1.4e-08		LOC441666	Body	Island
2:21266726_21266995	R0-NR0	NK	6	hypo	7.5e-09	APOB (+84)	APOB	TS200	Island
2:43903581_43903842	R1-NR1	Bcell	3	hyper	1.7e-08		PLEKHH2;LOC728819	TS200	OpenSea
6:1604012_1604212	NR1-NR0	Mono	5	hypo	1.3e-08				N_Shore
12:52473421_52473731	R1-NR1	Bcell	4	hyper	2.3e-08				Island
2:109746690_109746754	R1-R0	Neu	4	hyper	5.6e-09		LOC100287216;SH3RF3	TS200	Island

Region chr:bp	Model	Cell type	No. CpG	ΔMeth.	*p*	GREAT annotation[a] Gene (Dist. to TSS)	Illumina annotation Gene	Feature	CpG Island
19:54106592_54106876	NR1-NR0	Mono	3	hypo	2.5e-08		LOC284379	TS200	OpenSea
13:23309929_23310464	R1-NR1	Bcell	5	hyper	5.2e-08				OpenSea
16:54210495_54210550	R0-NR0	NK	2	hypo	6.5e-09				OpenSea
6:30419490_30419576	R0-NR0	Bcell	5	hyper	2.2e-08				Island
17:41438329_41438402	R0-NR0	CD8T	3	hyper	2.1e-08				Island
10:8096632_8096818	R1-NR1	Bcell	5	hypo	3.3e-07		FLJ45983	TS200	Island
11:117069848_117070046	R1-NR1	NK	4	hypo	5.5e-07		TAGLN	TS200	OpenSea
6:32847761_32847845	R0-NR0	NK	6	hypo	4.7e-06		PPP1R2P1	Body	Island
22:31318239_31318373	R1-NR1	Bcell	3	hypo	2.8e-05		C22orf27	TS200	Island
3:159557778_159557797	R1-NR1	NK	2	hypo	3.7e-05	SCHIP1 (+138)	SCHIP1	Body	OpenSea
2:113993051_113993142	R1-NR1	Bcell	2	hyper	5.2e-04		PAX8; LOC440839; LOC654433	Body;Body; Body;Body; Body;Body; TSS1500	N_Shore
6:31939279_31939322	NR1-NR0	Mono	3	hypo	3.6e-04		STK19;DOM3Z	TSS1500;TSS1500;Body; Body	N_Shore
4:103940922_103940950	R0-NR0	Bcell	2	hypo	2.8e-04		NHEDC1;NHEDC1	TS200	Island
6:32121112_32121204	R1-NR1	Neu	5	hypo	4.6e-03		PPT2;PRRT1	TS200	N_Shore
11:86383235_86383241	R1-NR1	Bcell	2	hypo	5.1e-03		ME3	TS200	Island
20:3052114_3052151	R1-R0	NK	2	hypo	4.2e-03	OXT (-133)	OXT	TS200	Island

Abbreviations: bp, base pair; chr; chromosome; CI, Confidence Interval; FDR, false discovery rate; GREAT, Genomic Regions Enrichment of Annotations Tool; Hyper, Hypermethylation; Hypo, Hypomethylation; NR, treatment non-responder; R, treatment responder; T_0, baseline visit.

[a] Distance from CpG to transcription start site of nearest upstream and downstream gene from rGREAT.

Table S-3. Results from analyses among self-report white participants for *limma* DMP results with $p < 1 \times 10^{-6}$.

		Primary Analyses		Within self-report white participants only	
**CpG**	**Model[b]**	**% ΔBeta**	***p***	**% ΔBeta**	***p***
cg06336912	T_1: R-NR	-5.86	6.5e-6	-6.49	6.2E-06
cg15936718	T_1: R-NR	-3.49	8.9e-6	-3.72	2.8E-05
cg16868591	$(R_1-R_0)-(NR_1-NR_0)$	-3.39	3.0e-6	-3.24	2.5E-05
cg19506849	R_1-R_0	2.64	9.4e-6	2.63	1.4E-06
cg18224793	NR_1-NR_0	2.34	4.7e-6	2.34	2.6E-06

Abbreviations: bp, base pair; chr; chromosome; CI, Confidence Interval; FDR, false discovery rate; GREAT, Genomic Regions Enrichment of Annotations Tool; NR, treatment non-responder; R, treatment responder; T_0, baseline visit.

[a] Distance from CpG to transcription start site of nearest upstream and downstre5 am gene from rGREAT.

Table S-4. Results from analyses among self-report white participants for *TCA* DMP analyses.

CpG	Model	Analysis	Cell type	Δ Meth.	Joint model	Marginal conditional model p-value
cg13249593	T_0: R-NR	Primary	CD4T	0.226	*p*: 2.4E-8<br>p_{FDR}: 9.5E-3	4.6E-6
			CD8T	-0.310		1.8E-5
			NK	-0.489		2.3E-6
		Within self-report white participants	CD4T	0.217	*p*: 4.8E-7<br>p_{FDR}: 0.957	3.8E-5
			CD8T	-0.315		5.0E-5
			NK	-0.501		2.6E-5

Abbreviations: FDR, false discovery rate; GREAT, Genomic Regions Enrichment of Annotations Tool; NR, treatment non-responder; R, treatment responder; T0, baseline visit.

a Distance from CpG to transcription start site of nearest upstream and downstream gene from rGREAT.

b Model comparing difference in DNA methylation baseline before MTX treatment between those were identified as treatment responders compared to non-responders.

c Joint model from tensor composition analysis tests for evidence of differential methylation within any cell-type at each CpG.

Table S-5. Meta analysis results for *limma* DMP results with $p < 1 \times 10^{-6}$.

		Primary Analyses		Meta-analyses [a]		
**CpG**	**Model[b]**	**% ΔBeta**	***p***	**% ΔBeta**	***p***	**p_Q [b]**
cg06336912	T_1: R-NR	-5.86	6.5e-6	-5.00	2.0E-08	0.84
cg15936718	T_1: R-NR	-3.49	8.9e-6	-5.58	7.4E-04	0.84
cg16868591	$(R_1-R_0)-(NR_1-NR_0)$	-3.39	3.0e-6	-4.36	1.5E-05	0.71
cg19506849	R_1-R_0	2.64	9.4e-6	2.38	9.5E-04	0.94
cg18224793	NR_1-NR_0	2.34	4.7e-6	1.95	2.7E-03	0.70

Abbreviations: bp, base pair; chr; chromosome; CI, Confidence Interval; FDR, false discovery rate; GREAT, Genomic Regions Enrichment of Annotations Tool; NR, treatment non-responder; R, treatment responder; T_0, baseline visit.

[a] Inverse variance-meta analysis with random effect, calculated using Meta R package.

[b] P-value for Cochran's Q heterogeneity test. I^2 estimated to be 0 for all CpGs in this table.
